# Development and feasibility testing of a physical activity intervention for youth with anxiety and depression: a study protocol

**DOI:** 10.1186/s40814-022-01010-6

**Published:** 2022-03-02

**Authors:** Arne Kodal, Fiona Muirhead, John J. Reilly, Gro Janne H. Wergeland, Paul Joachim Bloch Thorsen, Lars Peder Bovim, Irene Bircow Elgen

**Affiliations:** 1 Department of Child and Adolescent Psychiatry, Division of Psychiatry, Haukeland University Hospital, p.b 1400, N-5021 Bergen, Norway; 2 School of Psychological Science and Health, University of Strathclyde, Glasgow, Scotland; 3 Department of Clinical Medicine, Faculty of Medicine, University of Bergen, N-5020 Bergen, Norway; 4 Department of Health and Functioning, Western Norway University of Applied Sciences, Bergen, Norway

**Keywords:** Anxiety, Depression, Youth, Physical activity, Intervention

## Abstract

**Background:**

Anxiety and depressive disorders in children and adolescents are highly prevalent and account for more than half of all youth psychiatric disorders. Left untreated, anxiety, and depression lead to numerous detrimental outcomes, including reduced quality of life, psychiatric, and somatic comorbidity and even reduced lifespan. This puts a large strain on child and adolescent mental healthcare services (CAMHS) to provide effective treatments. However, even when provided the best evidence-based treatment, between 40–50% of patients continue to report significant symptom burdens. Thus, there is an immediate need for supplemental and/or new treatment approaches. Physical activity as a supplementary treatment may be such an approach. However, research investigating this approach within this population is scant. This protocol paper describes the development and feasibility trial of a physical activity-based intervention targeting anxiety and depressive symptoms in youth treated in CAMHS.

**Methods/design:**

The study is based on the UK Medical Council Research Framework (MRC) for developing and evaluating complex interventions. Feasibility and acceptability of the physical activity intervention (confident, active, and happy youth) will be evaluated in an uncontrolled open-label trial using qualitative and quantitative data. Twenty youths with anxiety and/or depressive symptoms will be recruited. Acceptability of assessment procedures, the intervention, and perceived benefits and barriers to participation will be assessed, and qualitative interviews with participants, caregivers, and referring specialists will explore contextual and practical factors associated with intervention delivery. Physical activity will be measured using the Actigraph GT3X+ monitor at baseline, and post-intervention and change in anxiety and depression will be assessed.

**Discussion:**

This study will contribute to the development of supplementary physical treatment interventions for youth with anxiety and depression in contact with CAMHS. The goal is to examine new avenues of treatment that ultimately may improve upon current treatment outcomes of anxiety and depression. This work will be in preparation for a future definitive randomized controlled trial (RCT) of this approach, in line with the MRC Framework.

**Trial registration:**

ClnicalTrials.gov, NCT05049759. Registered on August 19, 2021. Retrospectively registered.

**Supplementary Information:**

The online version contains supplementary material available at 10.1186/s40814-022-01010-6.

## Background

Internalizing disorders in the form of anxiety and depression are the most common mental health disorders among children and adolescents (hereafter youth), with a prevalence of 6.5% for anxiety disorders (CI 95% 4.7–9.1) and 2.6% for depressive disorders (CI 95% 1.7–3.9) [1, 2]. These disorders tend to develop early in life, and short- and long-term consequences include reduced quality of life, psychiatric and somatic comorbidity, disability, loss of education and/or work, suicide, and reduced life-span [3, 4]. According to the World Health Organization, anxiety, and depressive disorders are major health problems with large-scale societal and economic consequences (WHO, 2017). Anxiety and depression are among the top five causes of overall disease burden among youth in Europe [5].

Discouragingly, even when provided the best evidence based treatment, post-treatment remission rates for anxiety and depressive disorders in youth are just slightly above chance, e.g., 50% (mixed anxiety: 50.7% CI 45.3–56.2; depression: 53.2%, CI 34.6–70.9 [6]. Such suboptimal treatments have adverse consequences for the individual, but also place a large strain on treatment services, which are dependent upon sufficient capacity and effective treatment. If patients do not recover or experience relapse following treatment, these patients require further treatment, thus excluding new patients from gaining access to treatment. In light of the high prevalence of these disorders, short- and long-term consequences, and the suboptimal effect of current treatments, there is a pressing need for development of new and supplementary interventions to improve recovery rates [6–8].

In recent years, physical activity has gained traction as a promising area in effective mental health treatments [9, 10], and physical activity is identified as a key modifiable factor in people with mental illness [11]. Meta-analyses of the effects of physical activity in the treatment of anxiety and depression in adult populations indicate small to moderate effects [12–14]. This knowledge is now implemented in adult depression treatment recommendations such as those provided by The European Psychiatric Association [15]. The amount of research on the topic in paediatrics is substantially less [9, 16]; however, new evidence is starting to accumulate. Three recent meta-analyses investigating the effect of physical activity on youth depression found small to moderate effects [16–18]. Studies examining the effects of physical activity on anxiety disorders in youth are even fewer, with only one meta-analysis. In this meta-analysis, Carter et al. (2021) concluded that physical activity is potentially effective in reducing anxiety symptoms within non-clinical populations of youth, while evidence within clinical (treatment seeking) populations is insufficient to draw any clear conclusions. All these recent meta-analyses call for well-designed research with youth populations—particularly clinical youth populations [16–19].

The mechanisms by which physical activity interventions impact anxiety and depression are complex and multifaceted [20]. Limitations of previous research and/or mixed results have hampered attempts to tease out the specific intervention mechanisms required to cause change. However, the key recent meta-analyses do point to a few common ingredients across the intervention studies which had positive effects. These ingredients included supervised, aerobic-, and group-based activity of moderate-to-vigorous intensity, with participants engaging in activities multiple times per week over a time period of at least 6 to 8 weeks [16–19]. Questions concerning optimum dosage, type of activity, and energy expenditure are still unclear.

Focusing on the more specific mechanisms of effect of physical activity on anxiety and depression in youth, several biological, psychosocial, and behavioral factors are likely to be involved. Neurobiological mechanisms may include processes that are disrupted or dysregulated and potentially modulated by physical activity. Processes may include inflammatory and oxidative stress responses, neurogenesis, modulation of monoamines (e.g., serotonin), and HPA axis regulation [21, 22]. Hillman et al. found that a program targeting cardiorespiratory fitness resulted in increased neural underpinnings of attentional resources on tasks requiring improved inhibition and cognitive flexibility [23]. Deficits in inhibition and cognitive control are associated strongly with anxiety and depression in youth [24].

In regards to psychosocial processes, physical activity can increase opportunities for social interaction, social connectedness, and experiences of mastery and can lead to increased self-efficacy and independence [20, 25]. Problems and deficits within these areas are closely associated with anxiety and depression in youth [26, 27]. Exposure therapy is the treatment of choice for anxiety disorders in youth [6]. Similarly, physical activity is also assumed to affect anxiety and depression through exposure towards an avoided object and/or situation, i.e., physical activity itself induces bodily sensations and reactions that otherwise might be interpreted as symptoms of anxiety and/or be negatively appraised. Exposure to such sensations and reactions in this setting is thus assumed to be associated with non-threatening experiences, leading to normalization of such experiences and a more appropriate interpretation of these (Kandola et al. 2018).

In terms of behavioral mechanisms, physical activity may improve sleep, self-regulation, and coping skills [20]. The interplay of the mechanisms described above is most likely also highly idiosyncratic to the individual, based on their prior beliefs and experiences of physical activity.

### Development of a supplementary intervention

The development of a supplementary physical activity intervention took place in the Child and Adolescent Mental Health Services, Haukeland University Hospital, Bergen, Norway, from 2018 to 2020. A work and management group consisting of clinicians and researchers, and a trial steering committee consisting of stakeholders, clinicians, users, and parents identified key intervention components and a suitable delivery format. In addition, the group identified and outlined necessary organizational infrastructure, internal work procedures, and team composition in order to deliver the treatments effectively.

The target goals of the program link closely to interrelated core symptoms of anxiety and depression: reduced or low levels of physical activity [28], lack of confidence in one’s ability to cope with situations that incites distress and/or fear, and decreased willingness to engage in and avoidance of situations that may incite distress and/or fear [27, 29]. Finally, a core symptom particularly with depression is lowered mood. Taken together, the primary aim of the intervention is to alleviate these core symptoms and supplement ongoing treatment in CAMHS, which translates to helping youth become more *confident, active, and happy,* hence the name of the intervention*:* Confident, Active and Happy Youth (CAH-Y).

The primary aims of the intervention are as follows:

- Help youth become more confident, more happy and more physically active.
- Reduce symptoms of anxiety and depression.

Secondary aims of the CAH-Y intervention are as follows:

- To motivate and support youths with anxiety/depression symptoms to initiate and maintain an active lifestyle and reduce sedentary behavior.
- To help foster integration of physical activity into current treatment approaches for internalizing disorders in youth in treatment in CAMHS.

While the effects of the physical activity itself are important and required in the intervention, there is also a need to address underlying reasons why youth with anxiety and depression avoid and/or have difficulty doing physical activity. To this end, the CAH-Y intervention draws on Self Determination Theory [25] and Inhibitory Learning Theory [30]. Self Determination Theory provides a framework with which to understand participant experiences with physical activity and their *motivation* (as opposed to avoidance) towards physical activity. The framework focuses on ways to enhance learning and intrinsic motivation concerning both increased activity and behavior and symptom change [25]. More specifically, the intervention attempts to satisfy youths’ basic psychological needs for autonomy (e.g., providing youth with choice), competence (e.g., adapting exercises to meet the needs/fitness levels of students), and relatedness (e.g., promoting an inclusive group environment). Inhibitory learning theory provides a generic framework to understand the bi-directional interaction between cognition, affects, and behavior in regards to anxiety and depression; in particular the role of physical activity avoidance as a coping and affect-regulation strategy that both causes and maintains these disorders [30].

Drawing together these theories and known components, a treatment manual was developed detailing a supervised, therapist-led, group-based physical activity programme involving aerobic exercise bi-weekly over a course of 7 weeks. Further details on the intervention are detailed below in the *intervention* section. The full treatment manual (in Norwegian) is available on request by contacting the corresponding author.

In line with guidance from the UK Medical Council Research Framework (MRC) [31], the present study is an initial step in determining if the developed intervention is feasible and acceptable for youth with internalizing disorders in CAMHS. A mixed-methods approach was chosen to explore indications of participants’ response and view to the intervention and thereby gain a more full understanding of the intervention acceptability [32]. The present study also includes a calculation of effect size estimates for outcome measures to estimate the sample size of a future “definitive” randomized controlled trial (RCT) as defined by the UK MRC.

The principal aims of this study are as follows:

1. To quantify recruitment rate, willingness to participate, attendance, treatment retention, and adherence
2. To assess the appropriateness and practicality of the designed intervention in the proposed settings
3. To determine the acceptability of recruitment strategies and the intervention by participants and willingness to participate
4. To assess the appropriateness and acceptability of the assessment tools
5. To provide preliminary evidence of effects of the intervention on physical activity, mood, and anxiousness. This evidence will provide an initial indication of whether the intervention can contribute to change within this group. Estimated effect sizes will provide the parameters for a definitive randomized controlled trial.

## Methods and design

**Fig. 1:**
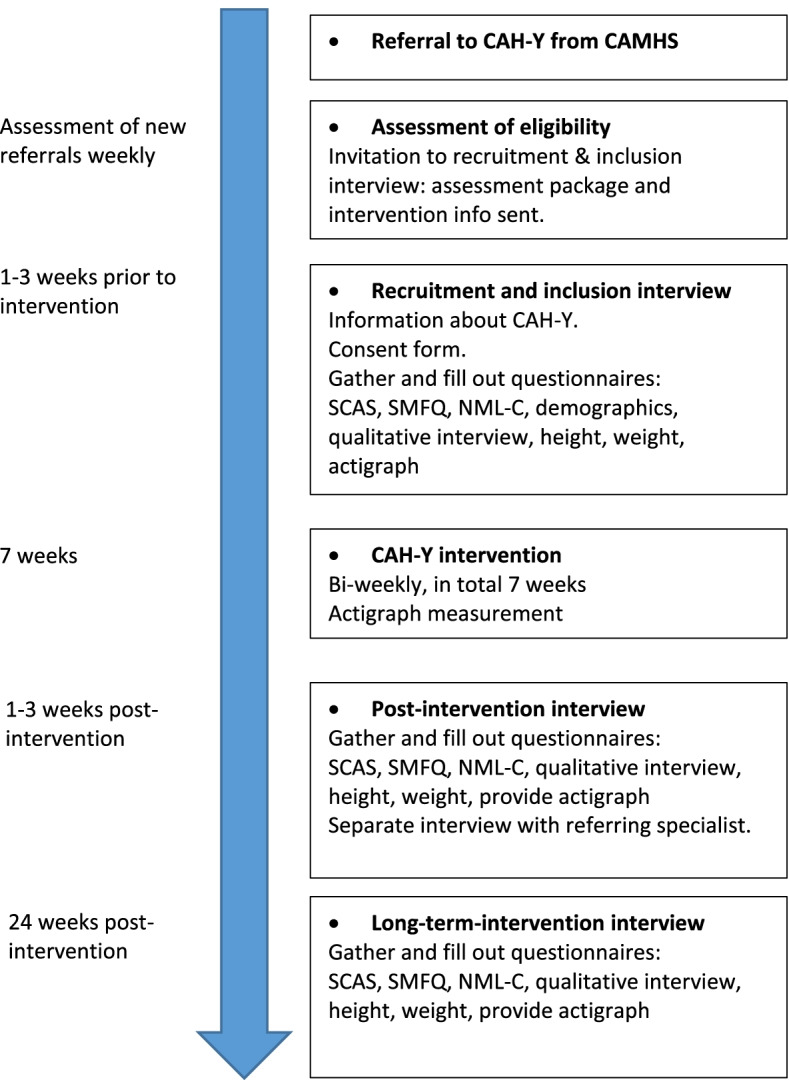
Study flow chart for CAH-Y feasibility study. *CAH-Y* Confident, Active and Happy Youth, *NML-C* Nijmegen Motivation List for Chldren, *SCAS-C/P* Spence Children’s Anxiety Scale, Children/Parents, *SMFQ* Short Mood and Feelings Questionnaire

This protocol conforms to guidelines presented in the Consolidated Standards of Reporting Trials (CONSORT) [33] 2010 statement extension for randomized feasibility studies and clinical trial protocols (see Additional file: Table S1). The intervention component is an uncontrolled open-label feasibility trial (hereafter referred to as “the intervention”), utilizing a pre-test–post-test within-subject design. The overall study design is illustrated in Fig. 1 (below), whereas the stages of the enrollment, interventions, and assessments Table 1 below (The SPIRIT table).

### Spirit table

**Table 1:** SPIRIT table for an uncontrolled open-label feasibility testing of physical activity intervention: Confident, Active and Happy Youth

Timepoint	Feasibility test period				
	Enrolment	Allocation	Intervention		Follow-up
	*T1*	0	*Weeks 1–7*	*T2*	*T3 (follow-up)*
Enrolment					
Eligibility screen	X				
Informed consent	X				
*Baseline assessment*	X				
Allocation		X			
Interventions					
*CAH-Y intervention*					
Assessments					
*Actigraph*	X		X	X	X
*Baseline data (demographic, psychiatric)*	X				
*Biometric data (height, weight)*	X			X	X
*Feasibility outcomes*	X		X	X	X
*SCAS-C/P, SMFQ, NML-C*	X			X	X
*Qualitative interview*	X			X	X

*CAH-Y* Confident, Active and Happy Youth, *NML-C* Nijmegen Motivation List for Chldren, *SCAS-C/P* Spence Children’s Anxiety Scale, Children/Parents, *SMFQ* Short Mood and Feelings Questionnaire

#### Intervention (CAH-Y)

The CAH-Y intervention includes two sessions of 50 min per week, for 7 weeks, in total 14 sessions. However, the final session is approximately 3-h long since it includes a nature hike and a conclusion of the program. Several constraints shaped the delivery format in the form of intervention length, intensity, and overall dosage. Thus, the delivery format is a compromise between evidence from behavior change research, organizational considerations, and clinical considerations regarding the logistical and practical burden on youth and parents (e.g., time spent to commute to treatment, time away from school). Importantly, despite these constraints the intended dose of the intended active mechanisms is still likely to be sufficient to have effect (e.g., moderate to vigorous activity, inhibitory learning, building motivation).

The intervention is group-based with up to eight participants in each group. Two therapists lead each group. Groups are divided into a child group and an adolescent group to accommodate maturational differences. Youth aged 8–13 receive the child intervention, whereas youth aged 14–18 receive the adolescent version. Both versions follow the same format and structure and have the same guiding principles and goals. However, the specific activities in CAH-Y 13 and CAH-Y 18 are tailored to the meet the maturational level and interests of the different age groups.

Each session follows the same structure and format (with exception of the last session as noted above). This structure consists of the following: an introduction and warm-up (10 min), main activity (35 min), and a session wrap-up (5 min). The separate sessions have distinct overarching topics and include a variety of activities. Sessions take place in a gym hall, two sessions involve activities in a swimming pool, and the last session is outside.

Concerning the more specific physical activities in the intervention, sessions include a mix of aerobic (e.g., running, jumping, traversing an obstacle course), resistance (e.g., squats, push-ups), and relaxation exercises (e.g., yoga exercises). The goal for each session is a minimum of 30 min spent in moderate to vigorous intensity activity.

Specifically during the introduction and warm-up, the therapists give psychoeducation to the youth regarding the bidirectional interplay between thoughts, feelings, actions, and particularly the role of avoidance and alternative coping strategies. The exercises are designed to stimulate some distress, fear, and/or discomfort, and the therapists couple these feelings and reactions to the introductory psychoeducation and use the exercises as exposure therapy. A supportive and mastery motivational climate facilitates the individuals need for relatedness [25] and a safe space to practice exposure (being observed), provides an optimal environment to help the youth address, and overcome this fear [34]. Thus, the group format allows youth to observe other youth, allowing them to observe that other youth have similar feelings, fears, reactions, behaviors, and responses as themselves, e.g., we sweat when we are physically active, trying out new activities may make us feel anxious but can be done nonetheless. Gaining this knowledge can help normalize the youth’s understanding of both themselves and others. Importantly, group and peer support are identified as an important intervention component to support active lifestyles [35, 36].

In accordance with self-determination theory, the sessions also strive to satisfy the youths’ needs for autonomy (e.g., giving them choices within a menu of activities to do), competence (e.g., adapting exercises to meet the needs/fitness levels of students), and relatedness (e.g., focusing on group cohesion, group cooperation). Importantly in this regard, the sessions do not have a competitive focus, but focus on being supportive, enjoyable, and fun. An overview of the CAH-Y intervention is presented in Table 2.

**Table 2:** Description of the intended structure and format of Confident, Active and Happy Youth program. Notes: *Pa* physical activity

Introduction Sessions 1–2	Treatment stage Sessions 3–12	Closing and transitioning Sessions 13–14
**Main theme:** Alliance and motivation Feeling secure in the group Alliance to the group/therapist Motivation to partake	**Main theme:** Challenge and exposure Moderate to vigorous PA From avoidance to exposure Strengthening motivation	**Main theme:** Transitioning to daily life Active “lifestyle” Continue exposure to PA Motivation to continue
Week 1 2 sessions of 50 min	Week 2-6 10 sessions of 50 min 2 sessions weekly	Week 7 1 session of 50 min 1 session of 3

*PA* Physical activity

### Therapists

Group-leaders will have knowledge of mental health disorders, youth development, group-dynamics, and physical activity to adequately understand and be able to meet the needs of the youth. Thus, a complementary team consisting of mental health nurse and a youth physiotherapist was recommended by the steering committee, to lead the intervention sessions. Additionally, the need to be able to rapidly address and intervene if youths experience discomfort or need adjustments to activities while still instructing the other youth, mean that at least two therapists are needed per session.

### Study population and eligibility

Youth are referred to the treatment program from their attending therapist at the local CAMHS. Youth can be included in the study if they meet the criteria below.

Inclusion criteria are as follows:

- Age 8–17 years
- Symptoms of anxiety and/or depression
- Youth display reduced daily physical activity (less than 30 mins per day and/or does not partake in physical leisure activities, and/or does not participate in physical education in school).
- The youth is motivated to partake in physical activity

Exclusion criteria are as follows:

- Physical activity is not advised for medical reasons
- Severe learning disabilities and the youth is unable to understand the study protocol
- Specific psychiatric disorders including any eating disorder, psychosis
- Severe challenging behavior or other needs requiring constant one to one support

### Recruitment

Youth will be recruited from the CAMHS, Department of Child and Adolescent Psychiatry, Haukeland University Hospital, Norway. CAMHS is organized at the same intervention level as ordinary hospital services (Haukeland University Hospital, Bergen, Norway) and is as such a tertiary specialized service. In the Norwegian context, this is the primary source of treatment for youth with mental health disorders. CAMHS is organized in three inpatient wards, three specialized clinics, and seven outpatient clinics. These units together form the recruitment base for CAH-Y. CAMHS offers all forms of mental health services to youth in a catchment area of app. 460,000 citizens in total. The CAMHS receives approximately 3000 referrals per year and delivers treatment to 5.2% of all youth in the catchment area [37]. Taking into account CAH-Y inclusion and exclusion criteria, we estimate that 40% of these youth qualify for participation in the intervention (*n* = 600). The likely numbers of patients referred and eligible should greatly exceed the numbers required by the feasibility study, meaning well below 10% of those eligible are required to consent.

Given that CAH-Y is organized within CAMHS, this enables easy access to potential participants and streamlined recruitment strategies. In practice, this translates into the following:

- Uncomplicated dispersion of information regarding the intervention to the departments and mental health professionals via intranet, email and/or physical/digital meetings
- Ease of implementing an in-house digital referral system
- A low threshold for communication between the CAH-Y team and the referring specialist

Based on the therapist’s assessment of eligibility and participant’s interest in the intervention, the therapist sends a formal referral to the CAH-Y team. The referrals are assessed with respect to inclusion/exclusion criteria by the Principal Investigator (PI is the first author). If the youth is assessed eligible, an invitation to attend a recruitment and inclusion interview is sent to the youth (and parents if the youth is below 16 years of age). Enclosed in the letter are also a number of health assessment questionnaires. The questionnaires assess mood, anxiety, demographic information, and motivation to attend the intervention. Finally, this letter informs of a webpage address, where the participant can find more information about the intervention and the study. The referring specialist will receive notification that the youth is eligible and is invited to an interview. Approximately a week prior to the interview, youth/parents are contacted via phone and they are given further information about the intervention and study participation.

### Consent

At the recruitment and inclusion interview, participants are provided further information about the intervention and the study, and any ensuing questions can be dealt with. Written information detailing the intervention, the study as well as the consent form are reviewed with the participant and/or parents if they participate in the interview. Informed written consent is obtained from all parents, and assent is obtained from youth above age 12 years. All participants in the intervention fill out the same questionnaires and go through the same assessment interview. Importantly, participation in the study requires written consent, but participation in the intervention is not dependent on participation in the study.

### Withdrawal of the study participants

At any time, participants can withdraw consent with no consequences for further intervention participation or other eventual benefits. If there are any serious adverse events, e.g., injury from the activities, details will be recorded and reported to the PI and to the work management group. CAMHS has appropriate insurance of the participants, if any adverse event should take place. In the case of adverse psychiatric effects are uncovered, this will be reported immediately to the participants referring specialist in CAMHS.

### Feasibility procedures

After referral assessment of eligibility, eligible youth and their primary caregivers are invited to attend a recruitment and inclusion interview. This interview will take place in the CAH-Y facilities. In the interview, therapists will provide further information about the intervention and study details, clarifying any questions and/or concerns, and obtaining written consent, and they collect and check the questionnaires included in the invitation for the recruitment interview. Moreover, additional questionnaires will be completed (see primary and secondary outcome measures section below), medical information gathered (height and weight), and participants will be provided an Actigraph activity monitor to be worn for the next 7 days. In addition, a short qualitative interview exploring participant and caregiver views on physical activity will be conducted. Finally, the youth and caregivers will be given a tour of the CAH-Y facilities in order to familiarize them with the setting.

The first session of the CAH-Y program will be between one to a maximum of 3 weeks after the interview. At this point, they will also return the Actigraph monitors and activity diaries. The intervention will then start. The day before every session, the participants will receive a SMS reminder of the session. After each session, participants are encouraged to visit a designated webpage for the program, where they can find further information about the next session. This is in order to remind the participants of the session clarify what is expected and encourage participation.

Following the last session of the intervention, participants will receive an invitation to attend a post-intervention interview, one to a maximum of 3 weeks following last intervention session. In this interview, questionnaires relating to symptom change will be completed, and biometric data will be gathered and a short qualitative interview exploring the views of the participant and his/her caregivers in relation to the intervention will be done. At the end of the interview, participants will be provided an Actigraph activity monitor to be worn for the next 7 days. After these seven days have elapsed, a research assistant will gather the Actigraph. Independently of this assessment with the participant, a short qualitative interview with the referring specialist will also be conducted by the PI.

At 6 months post-intervention, the participants and their caregivers will be invited to attend a follow-up interview. In this interview, questionnaires relating to initial symptoms will be completed, and a new short qualitative interview exploring views possible negative and/or positive effects of the intervention will be conducted. At the end of this interview, participants will be provided an Actigraph activity monitor to be worn for the next 7 days. After this time-period, the Actigraph will gathered by a research assistant. See Fig. 1 for study flow chart.

### Comparison group

Participants will serve as their own controls. Pre- and post-treatment results will be compared to inform a future power calculation.

### Measures

Apart from the measures that participants and their caregivers fill out at home [SCAS, SMFQ, demographics], all other measures and assessments will be conducted in the CAH-Y facilities.

### Socio-demographic characteristics and psychiatric conditions

Primary caregivers will provide socio-demographic information. The PI will acquire psychiatric/medical information, via the participant’s digital medical records. Axis-1 diagnoses are given during ordinary clinical practice by the youth’s psychiatrist or psychologist after reaching a consensus with other professionals from the multi-disciplinary team. In this study, we will classify the patients according to their main Axis I psychiatric diagnoses (ICD-10 codes are specified in Table 2) in the following groups: *depressive disorders*, *anxiety disorders*, *hyperkinetic disorders*, *pervasive developmental disorders,* and *other disorders* (a broad spectrum of psychiatric disorders with low frequency). The assessment and diagnosis of youth in CAMHS will provide the necessary information in order to asses study eligibility according to the predetermined inclusion/exclusion criteria.

### Feasibility outcomes

Feasibility of the trial will be done by way of continuous tracking of participant recruitment (number of youth referred/number of the youth assessed as eligible), attendance, and retention. The appropriateness, practicality, and acceptability of the intervention within the CAMHS setting, will be explored through feedback from participants, primary caregivers, and the referring specialists. This information will be gathered via semi-structured qualitative interviews, during the pre- and post-treatment assessment, while the referring specialists will be interviewed separately following the post-treatment assessment. Furthermore, the researchers will meet with the steering group at the end of the study to discuss the results and intervention acceptability.

### Participant-centered outcome measures

*Objective measures of physical activity* will be collected using the wearable activity sensor Actigraph GT3X+ monitor. The sensor captures and records continuous, high-resolution physical activity, and sleep/wake information and can record daily time spent in sedentary, light, and moderate to vigorous physical activity (MVPA). Other research has documented this method to be valid and reliable [38]. Pre-defined minimum wear time for a valid day will be defined as 6 h per day with a minimum of 3 days of data required for analysis inclusion [39]. Data for the Actigraph will be downloaded using Actigraph Actilife software and interpreted using 30-s epochs and the following cut-off points: sedentary (<100 cpm) and MVPA (≥3200cpm) [40]. Non-wear time will be defined as 60 min of consecutive zero counts. Participants will also be asked to wear the Actigraph during each intervention session to provide data on physical activity and MVPA content of the sessions.

Participant *height* will be measured in meters and rounded to the nearest 0.1 cm. *Weight* will be measured in kilograms and rounded to the nearest 0.1kg.

*Anxiety symptoms* will be assessed using Spence Child Anxiety Scale, child, and parent version (SCAS-C/P) (Spence, 1998). The SCAS comprises 38 items rated on a 4-point scale (0 = never, 1 = sometimes, 2 = often, 3 = always), with a maximum score of 114. SCAS-C/P has demonstrated good psychometric qualities [41].

*Affective symptoms* will be assessed using Short Mood and Feelings Questionnaire, and child and parent version (SMFQ-C/P) (Angold, Costello, Messer, & Pickles, 1995) is used to assess youth affective symptoms. The SMFQ has demonstrated good psychometric qualities [42].

*Intervention motivation* will be assessed using Nijmegen Motivation List Child (NML-C) [43]. This is used widely to assess treatment motivation in children and adolescents. The NML-C comprises 15 items rated on a 3-point scale.

The semi-structured qualitative interview will apart from feasibility outcomes also assess participant perceptions on PA in general, participant views on the association between PA and mental health, perceptions on the intervention components and the intervention as a whole, views on potential barriers to physical activity, motivation for activity, and participation in school-based physical activity. These questions will be posed at post-treatment and long-term follow-up. Caregivers will be posed the same questions; however, caregivers of youth aged 12 years or above will only receive these questions if the youth has consented to this. Answers to the questions are written down during the interviews and will be analyzed by thematic analysis [44]. An interview with the referring specialist, following the post-treatment interview with the participant, will similarly explore his/her experiences of the intervention including acceptability of procedures, perceived benefits, and difficulties.

An effect-size will be calculated for youth and caregiver reported depression and anxiety symptoms and activity level. Means (*M*) and standard deviations (*SD*) will be used to investigate the effect sizes for change between pre- to post-treatment. These results will help inform the power calculation of the likely required sample size for a future large-scale RCT.

### Timing

Measures will be assessed at baseline (pre-intervention interview) and at the latest 2 weeks after completing the intervention. Follow-up outcome will be assessed 6 months post-intervention.

### Sample size

As this will be the feasibility study to inform the design of the future definitive RCT, a target sample of 20 youth participants will be recruited for a mixed-methods assessment of feasibility [45].

### Progression criteria

A feasibility study is not adequately powered to test the any hypothesis (e.g., the efficacy of CAH-Y physical activity intervention) but is intended to improve the chances of conducting a high-quality RCT. Therefore, in line with suggestions by el-Kotob et al. [46], a priori criteria for progression to the definitive large-scale RCT are advisable. The progression criteria to move on to a definitive large-scale RCT will be as follows: (A) no serious adverse events, such as hospitalization, a life-threatening condition, death, and any adverse events associated with the intervention; (B) recruitment rate of no less than 75%; and (C) retention rate of no less than 60% in each group at 7 weeks (total of 14 sessions). If all the three criteria are not met, we assess there is insufficient evidence to justify proceeding to the definitive RCT. Consequently, changes to the intervention would then be required with consequent re-runs of the intervention following, up until these criteria are met.

### Statistics and data analysis

Quantitative data will be analyzed using SPSS version 22.0 for Windows (SPSS Inc., Chicago, IL).

Baseline data for participants will also be presented in charts as well as any possible participant missing data (questionnaires and Actigraph). Descriptive data will use 95% confidence intervals (mean and standard deviations). Baseline differences between groups (e.g., age, activity level, BMI, number of mental health disorders, and questionnaire outcomes) will be analyzed using one-way ANOVA. Nominal data (e.g., gender, ethnicity, social class, participation in school physical activity) will be analyzed using chi-square analyses. However, as mentioned, the feasibility study is underpowered to detect any effects reliably. Thus, quantitative feasibility outcomes will be interpreted only as feasibility and pilot data due to lack of statistical power.

Qualitative interviews with participants, caregivers and referring specialists will be analyzed utilizing thematic analysis [44]. Accordingly, the interview data will be reviewed for data familiarization; initial codes will be generated, followed by organizing codes into themes, refining themes, and finally defining themes and sub-themes.

### Possible harms

Potential harms of being involved in the intervention, and the assessments will be explicitly outlined in the participant explanatory statements and consent form. During assessment, the primary potential risk for participants may be to experience some psychological distress; however, this is not anticipated to exceed levels of psychological distress experienced previously in CAMHS or in their daily lives. During the intervention, the youth are expected to engage in physical activity with varying degrees of activity intensity. During such activities, the youth could potentially injure themselves or others physically, and/or they could experience some physio-psychological distress. All activities required in the intervention are preplanned and will be completed so as be to be as safe as possible, yet any movement (in a group) does necessarily entail some risk of injury. All accidents and injuries will be recorded and reported to the PI and the work management group. In addition, any adverse events or mental states observed among the participants (e.g., significant symptom deterioration, suicidal ideation or suicide attempt, reported or observed abuse and/or self-harm, excessive weight loss) will be monitored routinely throughout the study. Any such adverse events will be immediately reported back to the youth’s referring specialist, who is responsible for the youth’s mental health care. All study therapists and interviewers are experienced in working with youth with anxiety and depression and in responding to distress.

## Discussion

The aim of the current study is to develop and feasibility test a supplemental physical activity program for youth with internalizing disorders in regular treatment in child and adolescent mental health clinics (CAMHS). In light of high prevalence of anxiety and depression among children and adolescents, negative short- and long-term consequences, poor recovery rates even when provided best available treatment, there is an urgent need for new and/or supplemental treatment approaches. Physical activity may be one such approach. While the effects of physical activity on internalizing disorders in adults are to some degree empirically underpinned, the benefits, and supplemental treatment effects PA may have for youth are only just beginning to evolve. Thus, there exists a clear evidence gap in regards to the effects of PA on youth internalizing disorders and the integration of such an approach in CAMHS. A necessary first step in developing this field and addressing this knowledge gap is developing and feasibility testing a theoretically based physical activity-based intervention targeting anxiety and depressive symptoms in youth. This may be an important step towards addressing the burden and consequences these mental health disorders cause youth and to help provide more effective treatment.

We anticipate that participation in the intervention will result in increases in physical activity from pre- to post-treatment. Similarly, we also expect some improvements in anxiety and depressive symptomatology from pre- to post-treatment. These anticipated changes are hypothesized to map onto positive changes in youth confidence, mood, and physical activity. Concerning feasibility testing, we expect that feasibility estimates for recruitment, completion, and retention will be met, and results of outcome measures will enable an effect-size estimation for future RCT planning. It is anticipated that youth, parents, and referring specialists will find the intervention acceptable. The qualitative component will provide a unique opportunity to gain a rich perspective of the youths and parents experience and acceptability, which is essential towards future evaluation of the CAH-Y intervention.

### Limitations of the research

As this is a small-scale feasibility study with no control group, it is not possible to secure blinding throughout the study. Two therapists provide the recruitment and inclusion assessment interviews and deliver the intervention. However, some of the post-intervention assessments will be done by the study PI as well as the interview with the referring specialist and follow-up interview. Whilst a double-blind feasibility design would strengthen the study, time, and resources are limited and do not allow for this. However, in the next steps of the development of the intervention (in line with the MRC Framework); including a pilot RCT and the definitive RCT, necessary personnel to avoid bias in the trial will be secured.

### Strength of the research

The current study presents a number of strengths. Firstly, a major strength of the study is its alignment with the MRC Framework for the development of complex health interventions. The findings from this study are critical to the next stages of the MRC Framework: development and implementation of a definitive RCT exploring the effectiveness of physical activity as a supplemental treatment for youth with internalizing disorders in CAMHS. Secondly, a major strength of the study is a high degree of “real-world” or external validity. Many treatment development studies are based in university settings or specialized clinics and lead on to *efficacy* studies. The current study will both develop and aims to test effectiveness in a community setting in the longer-term, thus increasing is applicability in real-life settings and potentially bypassing any limitations and issues in generalizability from specialized to community clinic.

Thirdly, the target population for the intervention is a youth population which is especially susceptible to high levels of sedentary time and low levels of physical activity. Thus, this is a vulnerable target population in which changes in physical activity level can have a major impact both short-term and in regards to long-term health outcomes. Given that participants will give consent for access to medical journals, this will provide valuable insight into how comorbid illness influences feasibility, but also co-morbidity may be affected by the CAH-Y intervention.

### Trial status

The study is currently in progress, and participants are being assessed for eligibility and inclusion into the feasibility trial. Inclusion started in April 2020 and will continue until the target number of participants is included, likely before the end of 2021. Results from the study will be submitted for publication in the second quarter of 2022.

## Supplementary Information

**Additional file 1: Table S1**. CONSORT 2010 checklist for feasibility trial*. Notes: NA: Not applicable. (Eldridge et al. 2010).

## Data Availability

The treatment manual for CAH-Y is available on request (in Norwegian). Data sharing is not applicable to this article as no datasets were generated or analyzed.

## Abbreviations

ANOVA: Analysis of variance
CAMHS: Child and Adolescent Mental Health Care
CAH-Y: Confident, Active and Happy Youth
HPA: Hypothalamic–pituitary–adrenal axis
MRC: Medical Council Research Framework
MVPA: Moderate to vigorous physical activity
NML-C: Nijmegen Motivation List Child
PI: Principal investigator
RCT: Randomized controlled trial
SCAS-C/P: Spence Child Anxiety Scale, child and parent version
SMFQ-C/P: Short Mood and Feelings Questionnaire, child and parent version
